# The Foreign Journals: Midwifery

**Published:** 1841-01

**Authors:** 


### MIDWIFERY.

*Case of Fracture of the Skull occurring during difficult Parturition.*
By Dr. SCHULTZEN, of Insterburg.

A strong, healthy, and corpulent woman, about forty years old, had already had five children. All of them had been large, and all the births difficult, though accomplished without artificial assistance; two of the children were born dead. The sixth pregnancy was passed through well, and parturition came on at the right time. The pains were severe, and the membranes ruptured, but the delivery did not make progress. After twenty-four hours the author found the pains severe, and that much water had passed away, but that no blood had appeared. The uterus felt hard, and was contracted strongly round the child. The latter had a transverse position; the head lay above at the left side of the uterus, the feet below and to the right, the right hip presenting. The child was alive. The os uteri was completely open, the vagina very much dilated, and the turning of the feet was easily accomplished. The pelvis was, during this operation, ascertained to be of normal dimensions. After the turning, the body of the child was quickly forced down to the aperture of the pelvis by the continued strong pains, but here the head stopped with both arms lying close beside it, and with the face directed backwards and to the right, till at last with a further increase of the pains, and with the assistance of traction of the feet and shoulders, the child was quickly expelled. It gave no signs of life, and notwithstanding long-continued endeavours were made, it could not be revived. It exhibited nothing whatever abnormal externally, except a sugillation on the right parietal bone as large as a half-florin. It weighed a pound and three-quarters; its length was twenty-two inches and a half, the transverse diameter of the head three inches and three quarters, the long diameter four inches and three quarters, the breadth of the shoulders five inches. The bones of the head moved with great difficulty upon each other, and the anterior fontanelle was proportionally very small.

On examining the head, after its external coverings were removed, there appeared an extravasation of blood on the right parietal bone, amounting to about half a drachm. Directly below this the parietal bone was pressed in at its middle, and fractured in the form of a star. The indentation was of a round form, about an inch in diameter, three lines deep, and completely retained its shape when the bones were separated from one another. When held against the light the bones seemed very vascular, and it was evident that the fracture extended through both the tables. On the dura mater there lay a coagulum of blood of the size of a sixpence, and below it one rather larger. Another of the same size was found below the cerebellum, and the whole of the brain and its membranes were unusually vascular.

There could be no doubt that the death of the child was the consequence of this injury of the head; and it was equally certain that the injury was the result of the difficult parturition, though no instrumental means were employed.

*Casper's Wochenschrift. October* 10, 1840.

